# Intergenerational patterns of overweight and obesity among parents and preschool children: a population-based study in Sweden 2010-18

**DOI:** 10.1093/eurpub/ckag201

**Published:** 2026-11-08

**Authors:** Hasanka Kodagoda, Marie Lindkvist, Anneli Ivarsson, Sven-Arne Silfverdal, Masoud Vaezghasemi

**Affiliations:** Department of Epidemiology and Global Health, Umeå University, Umeå, Sweden; Department of Epidemiology and Global Health, Umeå University, Umeå, Sweden; Department of Epidemiology and Global Health, Umeå University, Umeå, Sweden; Department of Clinical Science, Pediatrics, Umeå University, Umeå, Sweden; Department of Epidemiology and Global Health, Umeå University, Umeå, Sweden

## Abstract

Childhood overweight and obesity (OWOB) are important public health concerns and parental BMI is associated with childhood adiposity. Less is known about the joint associations of maternal and paternal OWOB with childhood OWOB and obesity and this is the aim of this study. This population-based repeated cross-sectional study included 5937 three-year-old children and their parents from the Salut Programme in Västerbotten, Sweden, linked to national register data. Logistic regression was used to examine associations between parental BMI categories and child OWOB and obesity. Additive interaction between maternal and paternal obesity was further explored. The prevalence of OWOB among 3-year-old children was 15.3% including 2.4% with obesity. Maternal and paternal OWOB and obesity were associated with children’s OWOB and obesity, while no clear associations with parental socioeconomic or lifestyle factors were observed in this sample. Children with two parents with obesity had the highest observed prevalence of both outcomes. In adjusted analyses, the odds of child obesity were more than 3× higher when both parents had obesity than when neither had obesity. The findings indicate a possible positive departure from additivity. Parental OWOB and obesity were associated with children’s OWOB and obesity at 3 years of age, with the highest observed odds among children with two parents with obesity. No clear associations with parental socioeconomic or lifestyle characteristics were observed in this sample. These findings highlight an intergenerational pattern in weight status and the importance of considering parental weight status in early childhood obesity prevention.

## Introduction

Globally, overweight and obesity combined (OWOB) affect all population groups and remain a critical public health concern [1–3]. Between 1990 and 2022 the prevalence of OWOB among children and adolescents more than doubled, while obesity alone has quadrupled [1]. In Europe, OWOB has affected almost 60% of adults and one in every three children [4]. A systematic review 2019 [5] concluded that the pooled prevalence of OWOB in European pre-school children during the period 2006–16 was 17.9%. During the years 2016–20, an average of 12% of 4-year-old children in Sweden were overweight or obese [6]. The most recent study in the Västerbotten region in northern Sweden was conducted in 2025 among 4-year-olds, estimating that OWOB was more prevalent in girls than in boys (12% vs. 9.5%) in 2022. This study also reported a clear decline in the prevalence of OWOB since 2007 [7]. OWOB among adults is a public health concern in Sweden, impacting approximately 50% of the population [8]. Pre-pregnancy OWOB is common among expectant parents in the Västerbotten region, affecting about one-third of the women and half of the men [9–11].

Childhood obesity is associated with an increased risk of persisting obesity into adulthood and contributes to an increased risk of non-communicable diseases [3]. The causes of childhood OWOB are multifactorial and arise from complex interplay of genetic, biological, behavioural, social, and environmental factors [12] Children share genes with their parents but they also share family environment, dietary habits, physical activity patterns, and socioeconomic circumstances, all of which may contribute to similarities in body weight across generations [13–15]. Several studies have demonstrated intergenerational associations in health, including evidence that parental health status and health-related behaviours before and during pregnancy are associated with offspring health outcomes [16–18].

Parental body mass index (BMI) has consistently been identified as one of the strongest predictors of offspring BMI and obesity risk. Associations between parental and child BMI likely reflect a combination of genetic susceptibility, intrauterine influences, shared family behaviours, and common environmental exposures rather than any single mechanism. A recent systematic review and meta-analysis reported positive associations between parental BMI and BMI in adult offspring, with similar magnitudes of association for mothers and fathers and across sons and daughters [19]. Likewise, an evidence synthesis and predictive modelling study identified parental pre-pregnancy BMI as one of the most important predictors of BMI in mid-childhood [20].

Although parental BMI is a well-established predictor of childhood obesity, less attention has been given to the combined influence of maternal and paternal overweight and obesity. Examining parental BMI jointly may provide additional insights into the intergenerational patterns of overweight and obesity and help identify children at particularly high risk.

Therefore, the aim of this study was to examine the associations between individual and combined parental OWOB and obesity status before pregnancy and OWOB and obesity among children at three years of age, while considering parental socioeconomic and lifestyle factors.

## Methods

### Study design, setting, and data

This was a population-based repeated cross-sectional study which utilized data from 14 927 three-year-old children living in Västerbotten during 2014–18, along with their parents. The data were obtained from the Salut Programme which is an ongoing child health promotion and epidemiological surveillance programme integrated into the routine antenatal and child healthcare programme of the Västerbotten healthcare system.

The data obtained from the Salut Programme were based on three separate questionnaires that were filled out with nearly a 4-year time gap between them, corresponding to the time between early pregnancy and the child becoming 3 years of age. The first and second questionnaires were self-administered by the expectant mothers and fathers, respectively, and submitted at the first antenatal healthcare visit (gestational age 10–11 weeks). Data obtained were pre-pregnancy parental data on height and weight, place of residence, and lifestyle-related factors. For the mother, data on weight at the first antenatal healthcare visit were also collected. The third questionnaire was filled out by the parent or guardian when the child reached the age of 3 years and was returned at the child’s routine visit at the Child Health Care Centre. The child questionnaire was used to obtain data on age, sex, weight, and height to calculate the child’s BMI.

The 3-year-olds’ questionnaires and their corresponding pre-pregnancy parental questionnaires were filled out during 2014–18 and 2010–14, respectively. Statistics Sweden was responsible for linking the child data to their respective parental data using the personal identification numbers stated in each questionnaire and for obtaining parental data on country of birth, income, and education.

Figure S1 illustrates the initial study population of 10 689 three-year-olds and the steps in the exclusion of 4752 children due to the absence of consent, missing linking data, etc. The final sample consisted of 5937 three-year-olds with one or two parents each. The same dataset was used in another Salut study [21].

### Measures

The child’s and parents’ BMI values were calculated as weight in kilograms divided by height in metres squared. BMI categories were classified using WHO adult BMI cut-offs [3]. For the mother, both BMI before pregnancy and BMI at the first antenatal healthcare visit was calculated. Main analyses used the BMI before pregnancy for women; however, a sensitivity analysis used the BMI during pregnancy (gestational age 10–11 weeks). Child OWOB was defined using recently recommended extended International Obesity Task Force (IOTF) body mass index cut-offs, based on the international standard definition by Cole *et al.* [22]. These correspond with the relevant adult BMI cut-offs for normal weight (18.50 ≤ BMI ≤ 24.99), OWOB (BMI ≥ 25.00), and OB (BMI ≥ 30.00) categories (Table S1).

Two outcome variables were created using the child’s BMI categories: child OWOB and child obesity. These were dichotomized as OWOB vs. not OWOB, and obese vs. not obese. Children with BMI values classified as overweight or obese were grouped to form the OWOB category while only obese individuals were included in the obesity category.

A similar approach was applied to create the four exposure variables: mother’s OWOB status, father’s OWOB status, mother’s OB status, and father’s OB status. In addition to these individual parent variables, a joint-parental OWOB status variable was created, combining the parents’ binary individual OWOB variables and categorizing the combined parental outcomes as “neither OWOB,” “only father OWOB,” “only mother OWOB,” and “both OWOB.” The same approach was used to create the joint-parental obesity status variable.

In this study, individual parent responses retrieved from the separate questionnaires, or the data from Statistics Sweden, were combined to create independent variables on parental socioeconomic and lifestyle factors, using the same joint-parental categorization as in other publications from the Salut research group (Table S2). These variables included parents’ country of birth, parents’ education, parents’ income, parents’ alcohol habits, parents’ smoking habits, parents’ snuff use, parents’ drug use, parents’ sleeping habits and parents’ physical activity. In addition to these joint-parental variables we used the family’s place of residence and the child’s sex.

### Statistical analysis

Descriptive results were presented using frequencies and percentages together with Chi-square tests to assess the association between 3-year-olds’ OWOB or obesity outcomes with the explanatory variables. The relation between mothers’ and fathers’ OWOB and obesity status was further illustrated with frequencies, percentages and Chi-square tests. Multiplicative interaction between mothers’ and fathers’ OWOB and obesity status in relation to the children’s OWOB and obesity status was analyzed with logistic regression including a cross term. Bar diagrams were used to illustrate the relation between joint parental and child OWOB and obesity status. Multiple logistic regression analyses were used to analyse the association between joint-parental BMI status and the outcomes of child OWOB and obesity status in the presence of different socioeconomic and lifestyle factors. The results were presented as odds ratios (OR) with corresponding 95% confidence intervals (CI). Separate logistic regression analyses were not performed for the two sexes of the children since no interactions were found between sex and joint parental BMI status in relation to child OWOB and obesity status.

The Relative Excess Risk due to Interaction (RERI) arising from the interplay between parents’ joint OWOB or obesity status was further calculated. RERI_OR_ is an advocated measure to calculate the additional odds resulting from the additive interaction in a logistic regression environment and is calculated using the formula of RERI_OR_ = OR_11_ − OR_10_ − OR_01_ + 1 [23]. In this study, the RERI_OR_ values illustrate the excess (or deficit) odds of the child’s OWOB or obesity when considering the additive interaction between parental OWOB or obesity categories in relation to the outcome [24]. When the estimated odds of a child’s OWOB or obesity when both parents had either OWOB or obesity (OR_11_) differ from the sum of the estimated individual parental ORs (OR_10_ and OR_01_), the resulting excess or deficit odds are the impact of possible additive interaction (RERI_OR_).

Sensitivity analyses were conducted using mothers’ OWOB at the first antenatal healthcare visit for comparative multiple logistic regression analyses. All the analyses were performed using IBM SPSS Statistics Version 28.0.1.0.

### Ethics

The Regional Ethical Review Board in Umeå, northern Sweden, approved the study (2013-268-310 and 2017/40132). Only children whose parents had given written informed consent were included in this study, which was carried out in accordance with the ethical principles stated in the Helsinki Declaration of 1975 (as revised in 1983).

## Results

In total, data from 5937 three-year-olds and their parents were utilized in the study. A majority, 58.7%, of families lived in the most populous area of the region. Among the children, 87.7%, had Swedish-born parents and 2.2%, had both parents born outside Sweden. About one-third of the children had both parents with higher education than high-school and one-third of the children had both parents without higher education (Table S2).

### BMI characteristics of children and parents

Children’s and parents’ prevalence of OWOB and obesity are given in Table 1. The prevalence of children’s OWOB and obesity was 15.3% and 2.4%, respectively. Fathers had more than 20% higher OWOB prevalence (53.4%) compared to mothers (29.1%), while fathers’ obesity prevalence (10.5%) was closer to mothers’ (8.8%). The child’s BMI category and sex were not associated (Table 1). Mothers’ and fathers’ OWOB and obesity status were strongly associated with children’s OWOB and obesity status. If the mother or father had obesity, the prevalence of child OWOB increased from 14.1% to 23.8%, and the prevalence for child obesity doubled with mother’s obesity and almost tripled with father’s obesity. Further, mothers’ and fathers’ OWOB and obesity prevalences were strongly associated (Table 1). If the father was obese, 24.1% of mothers were also obese, compared to 7.1% if the father was not obese.

**Table 1:** Three-year-old children’s overweight/obesity (OWOB) or obesity related to parental pre-pregnancy body mass index, demographic and socioeconomic and lifestyle factors.^a^

	Child outcome				Relation mother and father			
Variables, *n* (%)	Child OWOB 907 (15.3)		Child obesity 143 (2.4)		Mother OWOB		Mother obesity	
	*n* (%)	*P* value	*n* (%)	*P* value	*n* (%)	*P* value	*n* (%)	*P* value
Mother OWOB								
No	451 (12.6)		66 (1.8)					
Yes; 1468 (29.1)	304 (20.7)	<.001	51 (3.5)	<.001				
Father OWOB								
No	288 (12.7)		37 (1.6)		427 (21.5)		107 (5.4)	
Yes; 2610 (53.4)	450 (17.2)	<.001	75 (2.9)	.004	781 (35.0)	<.001	261 (11.7)	<.001
Mother obesity								
No	649 (14.1)		99 (2.1)					
Yes; 445 (8.8)	106 (23.8)	<.001	18 (4.0)	.011				
Father obesity								
No	616 (14.1)		85 (1.9)		987 (25.9)		268 (7.0)	
Yes; 512 (10.5)	122 (23.8)	<.001	27 (5.3)	<.001	221 (53.3)	<.001	100 (24.1)	<.001
Sex of the child								
Boy	458 (15.0)		74 (2.4)					
Girl	449 (15.5)	.581	69 (2.4)	.921				
Place of residency								
Södra Lappland (rural)	95 (15.3)		17 (2.7)					
Skellefteå (hybrid rural/urban)	266 (14.5)		54 (2.9)					
Umeå (urban)	546 (15.7)	.523	72 (2.1)	.119				
Parents’ country of birth								
Both born in Sweden	788 (15.2)		122 (2.4)					
One born in Sweden	90 (15.2)		16 (2.7)					
Both born abroad	25 (18.9)	.500	5 (3.8)	.516				
Parents’ education								
Both more than a high-school education	325 (16.0)		41 (2.0)					
One more than a high-school education	301 (15.2)		53 (2.7)					
Neither more than a high-school education	281 (14.7)	.513	49 (2.6)	.348				
Family income								
Quintile 1 (highest)	192 (16.1)		29 (2.4)					
Quintile 2	165 (13.9)		26 (2.2)					
Quintile 3	193 (16.3)		28 (2.4)					
Quintile 4	188 (15.9)		36 (3.0)					
Quintile 5 (lowest)	169 (14.3)	.322	24 (2.0)	.555				
Parents’ alcohol habits								
Both non-risky habits	816 (15.5)		132 (2.5)					
One with risk use or addiction	72 (13.4)		9 (1.7)					
Both with risk use of addiction	14 (13.6)	.405	1 (1.0)	.328^b^				
Parents’ smoking habits								
None use snuff	720 (15.3)		102 (2.2)					
One use snuff	74 (14.9)		17 (3.4)					
Both use snuff	6 (19.4)	.796	1 (3.2)	.160^b^				
Parents’ snuff use								
None smoke	594 (15.8)		88 (2.3)					
One smoke	219 (14.4)		38 (2.5)					
Both smoke	11 (11.2)	.243	2 (2.2)	.917				
Parents’ drug use								
Both never used drugs	756 (15.3)		115 (2.3)					
One used drugs once or more	117 (15.4)		23 (3.0)					
Both used drugs once or more	27 (13.0)	.656	5 (2.4)	.503				
Parents’ sleeping habits								
Both 7 hours or more/day	601 (15.1)		92 (2.3)					
One less than 7 hours/day	245 (15.8)		41 (2.6)					
Both less than 7 hours/day	42 (15.3)	.801	10 (3.6)	.333				
Parents’ physical activity								
None less than 150 minutes/week	465 (15.7)		66 (2.2)					
One less than 150 minutes/week	316 (14.8)		57 (2.7)					
Both less than 150 minutes/week	115 (14.9)	.679	16 (2.1)	.490				

a Pearson’s Chi-square test was used to calculate the *P* values.

b Exact test.

### Socioeconomic characteristics

No clear associations were observed in this sample between parents’ socioeconomic or lifestyle characteristics and either children’s OWOB or obesity (Table 1).

### Children’s OWOB and obesity prevalence increased as parental BMI increased

Both OWOB and obesity prevalence of children increased if one or both of their parents had OWOB or obesity (Fig. 1). The prevalence of children’s OWOB and obesity were 30.6% and 7.1%, respectively, if both parents had obesity compared to 10.6% and 1.4% if neither parent had OWOB (Fig. 1 and Table 2). Table 2 further presents the results of multiple logistic regression models for the association between joint parental OWOB and obesity status and their children’s OWOB and obesity, adjusting for socioeconomic and lifestyle factors. None of the adjusting variables had a significant relation to children’s OWOB or obesity.

**Figure 1:**
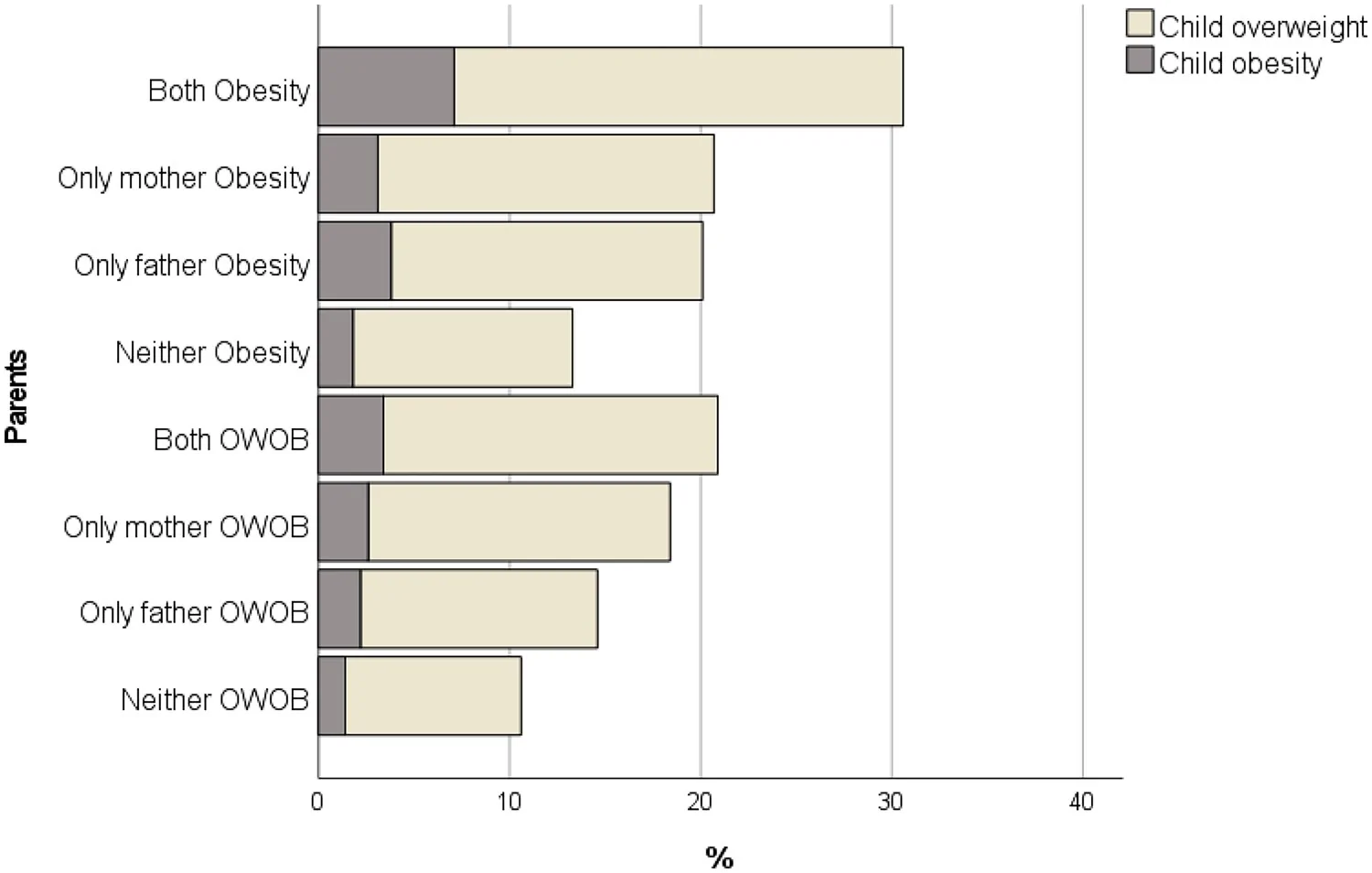
Bar diagrams illustrating the relation between joint parental and child overweight/obesity (OWOB) and obesity status.

**Table 2:** Associations of 3-year-old children’s overweight and obesity (OWOB) or obesity with parental combined OWOB and obesity status. Both crude and adjusted logistic regression analyses were used to calculate the odds ratios.

Parental BMI category combinations	*n* (%)	Child OWOB			Child obesity		
		*n* (%)	Crude OR (CI)	Adj OR^a^	*n* (%)	Crude OR (CI)	Adj OR^a^
OWOB within parents							
Neither OWOB	1534 (36.9)	162 (10.6)	1	1	21 (1.4)	1	1
Only father OWOB	1429 (34.4)	209 (14.6)	1.45 (1.16–1.81)	1.43 (1.13–1.80)	32 (2.2)	1.65 (0.95–2.88)	1.26 (0.70–2.27)
Only mother OWOB	423 (10.2)	78 (18.4)	1.91 (1.43–2.57)	1.98 (1.44–2.71)	11 (2.6)	1.92 (0.92–4.02)	1.63 (0.75–3.54)
Both OWOB	770 (18.5)	161 (20.9)	2.24 (1.76–2.84)	2.44 (1.88–3.16)	26 (3.4)	2.52 (1.41–4.51)	1.97 (1.05–3.69)
RERI			−0.12	0.03		−0.05	0.08
*P* value^b^		<.001			.016		
Obesity within parents							
Neither obesity	3484 (83.8)	463 (13.3)	1	1	63 (1.8)	1	1
Only father obesity	313 (7.5)	63 (20.1)	1.64 (1.23–2.20)	1.74 (1.27–2.38)	12 (3.8)	2.16 (1.16–4.06)	1.90 (0.94–3.83)
Only mother obesity	261 (6.3)	54 (20.7)	1.70 (1.24–2.33)	1.75 (1.23–2.49)	8 (3.1)	1.72 (0.81–3.62)	1.45 (0.61–3.47)
Both obesity	98 (2.4)	30 (30.6)	2.88 (1.85–4.47)	3.01 (1.87–4.84)	7 (7.1)	4.18 (1.86–9.37)	3.51 (1.43–8.65)
RERI			0.54	0.52		1.30	1.16
*P* value^b^		<.001			<.001		

a Adjusted for sex of the child, and parental demographic, socio-economic, and lifestyle factors.

b Pearson Chi-square test.

### Odds of child OWOB

In the adjusted model with joint parental OWOB status as the main exposure and child OWOB status as the outcome, families with only fathers being OWOB had 1.4× higher odds (*P < .*01) of the child having OWOB status, families with only mothers being OWOB had 2.0× higher odds (*P < .*001) and both parents being OWOB had an increased OR of 2.4 (*P < .*001) compared to when none of the parents had OWOB status (Table 2). In the adjusted model with joint parental obesity status as the main exposure and child OWOB status as the outcome, similar significant associations were found with even higher odds compared to the reference category (i.e. none of the parents having obesity status) (Table 2).

### Odds of child obesity

In both models with joint parental OWOB or obesity status as the main exposure and child obesity status as the outcome, the association between mothers’ and fathers’ separate effects on the outcome showed less significance (Table 2). However, the associations with both parents being either OWOB or obese remained strong and significant. The adjusted odds for a child having obesity status were almost two-fold (*P < .*05) if both parents had OWOB status compared to when none of the parents had OWOB status. The adjusted OR for a child having obesity status if both parents had obesity status was more than three-fold (*P < .*01) compared to when none of the parents had obesity status.

### Additive interaction

Figure 2 presents the RERI_OR_ estimates for the joint associations of maternal and paternal obesity with child OWOB and obesity. In the adjusted analyses, the RERI_OR_ point estimate was 0.52 for child OWOB and 1.16 for child obesity. Both estimates were positive and thus consistent with a possible positive departure from additivity, with a larger point estimate observed for child obesity.

**Figure 2:**
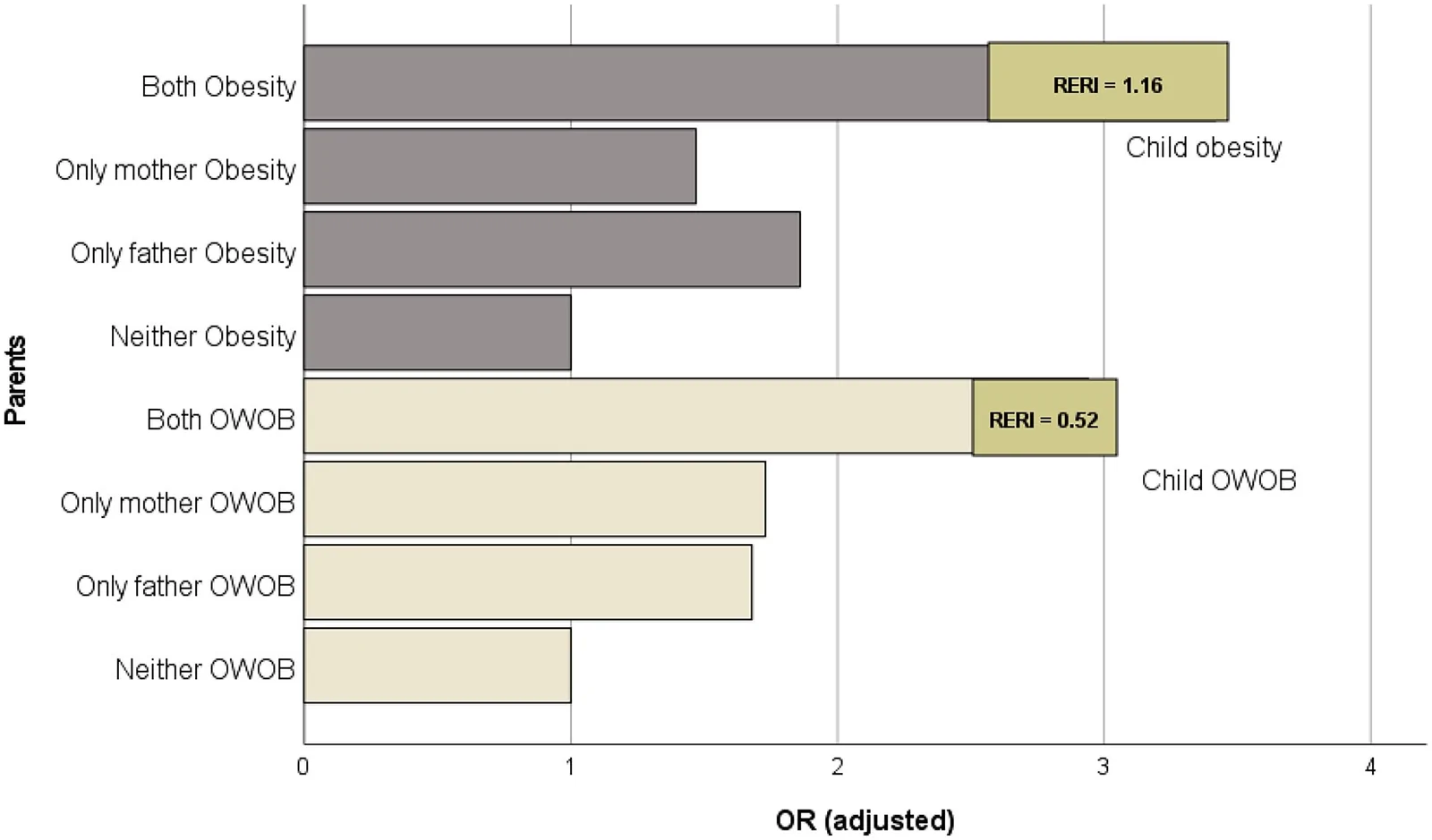
Odds ratio for child overweight/obesity (OWOB) and obesity according to maternal and paternal OWOB and obesity status. The relative excess risk due to interaction based on odds ratios (RERI_OR_) illustrates the estimated departure of additivity on the odds ratio scale. Estimates are adjusted for child sex and parental demographic, socioeconomic, and lifestyle factors.

### Sensitivity analysis

The sensitivity analysis, which replaced the pre-pregnancy maternal BMI variable with early pregnancy maternal BMI variable in analyses between joint-parental BMI status and the outcomes child OWOB and OB status resulted in results mostly consistent with the main analyses (Table S3). The largest difference in odds ratios was seen in the effect on only mothers’ OWOB association with child obesity. When early pregnancy BMI for the mother was used in the analysis, the association between mothers’ OWOB status and child obesity became stronger.

## Discussion

The study yielded some main findings. The OWOB prevalence among children was 15.3%, with 2.4% classified as obese. The difference between boys and girls regarding the prevalence of OWOB was marginal and regarding obesity non-existent. Further, 53.4% and 29.1% of expectant fathers and mothers, respectively, had OWOB status before the pregnancy and corresponding values for obesity status were 10.5% and 8.8%. None of the parental demographic, socioeconomic, and lifestyle factors were significantly associated with children’s OWOB or obesity in this sample. Both parents’ pre-pregnancy OWOB and obesity status were significantly associated with children’s OWOB and obesity. The prevalence of children’s OWOB and obesity increased with each parental OWOB and obesity combination, from neither being OWOB to both being obese. The prevalence and odds of child OWOB and obesity were highest when both parents had OWOB or obesity, with particularly high odds of child obesity when both parents had obesity. Positive RERI point estimates were observed for the joint association of maternal and paternal obesity with child OWOB and obesity, consistent with a possible positive departure from additivity.

The 3-year-olds’ OWOB and obesity prevalence in our current study approximates the levels found in a study involving 3- to 5-year-olds in some other regions of Sweden which for the years 2015–23 reported OWOB at 14.9% and obesity at 2.3% [25]. The OWOB prevalence for 4-year olds recorded in the Västerbotten region in 2009 [26] aligns with our current findings for boys, while girls’ prevalence was lower in our study. Interestingly, recent studies with a national sample of 4-year olds showed a somewhat lower OWOB rates (13.3% and 11.0%) [27, 28] compared to the current study’s findings.

The prevalence of OWOB among girls in the current study aligns with the 2018 prevalence among 2- to 6-year-old Finnish girls [29], while Finnish boys had a slightly lower prevalence (12%). In contrast, a Norwegian study with data from 2014 to 2016 reported a lower OWOB prevalence (10.8%) among 2- to 5-year-old children [30]. The OWOB prevalence in Västerbotten is lower than the European regional estimates of 17.9% OWOB among 2- to 7-year children during 2006–16 [5]. Additionally, the 2.4% child obesity prevalence in Västerbotten is considerably lower than the 12.7% obesity prevalence among 2- to 5-year-olds in the United States (data from 2017 to 2020) [31].

The current study’s findings on OWOB and obesity prevalence among expectant parents align with 3 other Swedish studies conducted in 2011 [11], 2013 [9], and 2024 [10]. Compared with adults in England and Finland, the prevalence of OWOB among expectant parents in the present study was lower. In a European comparison based on 2010 data, approximately 65% of English men and 62% of Finnish men, and around 47%–48% of women in both countries had OWOB, compared with 53.4% of fathers and 29.1% of mothers in the present study [32]. Likewise, the obesity prevalence in those two countries was nearly two-fold (around 20%) compared to the prevalence (around 10%) among Västerbotten adults [32]. The total OWOB rate (≈40%) in this study with mean parental age of 30 years, is lower compared to the average prevalence of 60% in the WHO European region [3] and the 48% average estimated for 18 European countries based on 2010 data [32]. Furthermore, the obesity prevalence among expectant parents in the current study (around 10%) was lower than the global average of 13% [3].

The current study found a strong association between pre-pregnancy OWOB of mothers and fathers and children’s prevalence of OWOB or obesity. Previous studies, including two meta-analyses [33, 34] and the Western Australian Pregnancy Cohort Study [35] have also reported similar associations. This is consistent with the findings of Öhlund *et al.* [36], which revealed a significant association between the father’s BMI and their 4-year-olds’ BMI. However, maternal and paternal BMI are not independent characteristics. They are strongly correlated, also shown in this paper and earlier research has discussed assortative mating [37] and shared living conditions which results in an environment where dietary habits, physical activity, parenting practices, and socioeconomic circumstances are shared. Parental BMI is likely to represent a marker of multiple interacting processes that jointly contribute to childhood overweight and obesity. The current study demonstrates clear associations between combined parental OWOB and obesity and the children’s OWOB and obesity, highlighting the importance of considering intergenerational patterns. These findings further emphasize the importance of family perspective in addressing childhood overweight and obesity. Similar findings and their implications have been discussed by other researchers [18, 24].

Moreover, the RERI_OR_ point estimates were positive for both child OWOB and child obesity, with a larger estimate for child obesity. Although this pattern is consistent with a possible positive interaction on the additive scale between maternal and paternal obesity, the point estimates alone do not establish the presence of additive interaction and should therefore be interpreted cautiously. Importantly, irrespectively of whether positive additive interaction can be established, children with two parents with obesity had the highest observed odds of obesity. In the adjusted analysis, the odds of child obesity were more than 3× higher when both parents had obesity compared with when neither parent had obesity, whereas the associations were weaker when only one parent had obesity. Thus, the findings demonstrate a clear pattern of higher observed odds among children with two parents with obesity, independently of the interpretation of the RERI estimates.

RERI point estimate for child obesity may suggest that the intergenerational pattern is more pronounced at the more severe end of the BMI spectrum. This is in line with Lindkvist *et al.* [24], who, in a sample of 18-month-old children reported higher odds of child OWOB when both parents had OWOB compared with when only one parent had OWOB. Together, these findings highlight the importance of considering childhood OWOB and overall health from a broader family perspective [18], particularly given the clustering of OWOB across generations [38]. Although the present finding do not provide evidence regarding underlying biological or causal mechanisms, the observed joint associations may be relevant from a public health perspective. From a prevention perspective, these findings support the relevance of considering the broader family context in health promotion and obesity prevention. Parental BMI may contribute useful information within a broader assessment of familial and individual circumstances, but the present study does not provide evidence for using parental BMI alone to determine which families should be prioritized for intervention.

The current study observed no clear association between parental demographic, socioeconomic, or lifestyle factors during the pre-pregnancy period and the child’s overweight or obesity outcomes at age 3 years. In contrast, a review of systematic reviews [39], and a review study based on 4 European cohort studies [40], have linked different unfavourable maternal lifestyle factors in the pre-pregnancy period with the risk of childhood obesity. Nevertheless, a somewhat similar finding to the current study was reported by Lindkvist *et al.* [24] in Sweden focusing on the association between family’s demographic and socioeconomic factors and the toddler’s BMI and at the child’s age of 18 months. Sweden’s high rate of preschool attendance among 1-to-5-year-olds [21] promotes shared common daytime meal and activity habits, potentially overshadowing the influence of societal inequalities. Furthermore, the 3-year lifespan analyzed in this study may be too short to fully capture the impact of societal inequities on young children.

### Methodological considerations

The study’s enrolment of the entire Västerbotten region enhances the generalizability of the findings to the Västerbotten region, Sweden and possibly to Scandinavian populations. The accuracy of OWOB and obesity prevalence estimates among children was high as trained health professionals measured children’s weight and height. Additionally, successfully linking on an individual level, three different questionnaires completed during different time frames and enriching these with Swedish government data was another strength of the study.

However, the study also has some limitations. Out of the full sample of participating 3-year-olds, 44% had to be excluded from the linking of data on an individual level, as was required for the analyses performed. This was due to missing or inaccurate personal identification numbers of either parents or the child or both, or due to missing or mismatched data, or the absence of parental consent. However, the children and parents included and excluded, respectively, had corresponding profiles with respect to core characteristics (data not shown), thus, the results can be considered representative of the population investigated. The reliance on self-reported data from mothers and fathers weight posed a potential limitation. Conversely, the sensitivity analysis using early pregnancy measurements provided additional confidence in the validity of the results.

## Conclusion

OWOB among preschool children and their parents remain key public health concerns in Västerbotten. Parental OWOB was strongly associated with child BMI, with combined maternal and paternal obesity associated with higher odds of child OWOB and obesity. No clear associations were found with parental socioeconomic or lifestyle factors in this study, highlighting the need for further follow-up research when children grow up. The consistency with international studies indicates that intergenerational patterns of OWOB are evident in parental characteristics observed before pregnancy. The observation that these associations are already evident at 3 years of age underscores the importance of early childhood as a period for obesity prevention. The findings highlight the potential value of considering parental weight status within existing maternal and child health services to support early identification of children who may be at increased risk of OWOB.

## Supplementary Material

ckag201_Supplementary_Data

## Data Availability

The authors do not have permission to share the data. Key points Overweight and obesity among preschool children continue to be an important public health concern in the Västerbotten region and beyond. Parental individual and combined overweight and obesity significantly increase the odds of overweight and obesity already in preschool age. Children with two parents with obesity had the highest observed odds of obesity, highlighting the relevance of considering the broader family context in childhood obesity prevention.
