# Supplementary material for: Intergenerational patterns of overweight and obesity among parents and preschool children: a population-based study in Sweden 2010-18
Source: Eur J Public Health. 2026 Nov 8;36(6):ckag201. doi: 10.1093/eurpub/ckag201 (PMC13744698; doi:10.1093/eurpub/ckag201)
Supplement: Supplementary file 1 [file ckag201_supplementary_data.zip › ejph-2026-01-om-0044-File004.docx]

| Life stage | BMI category | | | | | | |
| --- | --- | --- | --- | --- | --- | --- | --- |
|  | Underweight |  | Normal weight |  | OWOB |  | OB |
| Adult^a^ | BMI ≤ 18.49 |  | 18.50 ≤ BMI ≥ 24.99 |  | BMI ≥ 25.00 |  | BMI ≥ 30.0 |
| 3-year-old boys^b^ | BMI ≤ 14.82 |  | 14.83 ≤ BMI ≥ 17.84 |  | BMI ≥ 17.85 |  | BMI ≥ 19.50 |
| 3-year-old girls^b^ | BMI ≤ 14.59 |  | 14.60 ≤ BMI ≥ 17.63 |  | BMI ≥ 17.64 |  | BMI≥ 19.38 |

^a^ WHO (2021) global BMI cut-offs for adults ^b^ IOTF child BMI cut-offs for 3-years-olds that correspond with adult BMI cut-offs.
