# Supplementary material for: Intergenerational patterns of overweight and obesity among parents and preschool children: a population-based study in Sweden 2010-18
Source: Eur J Public Health. 2026 Nov 8;36(6):ckag201. doi: 10.1093/eurpub/ckag201 (PMC13744698; doi:10.1093/eurpub/ckag201)
Supplement: Supplementary file 1 [file ckag201_supplementary_data.zip › ejph-2026-01-om-0044-File005.docx]

| Variables n (%) |  | **Missing** n(%) |
| --- | --- | --- |
| Child characteristics |  |  |
| Child OWOB |  | 0 (0) |
| No | 5794 (97.6) |  |
| Yes | 143 (2.4) |  |
| Child Obestity |  | 0 (0) |
| No | 5030 (84.7) |  |
| Yes | 907 (15.3) |  |
| Sex of the child |  | 0 (0) |
| Boy | 3048 (51.3) |  |
| Girl | 2889 (48.3) |  |
| Parents’ BMI |  |  |
| Mother OWOB |  | 886 (14.9) |
| No | 3583 (70.9) |  |
| Yes | 1468 (29.1) |  |
| Father OWOB |  | 1051 (17.7) |
| No | 2276 (46.6) |  |
| Yes | 2610 (53.4) |  |
| Mother Obesity |  | 886 (14.9) |
| No | 4606 (91.2) |  |
| Yes | 445 (8.8) |  |
| Father Obesity |  | 1051 (17.7) |
| No | 4374 (89.5) |  |
| Yes | 512 (10.5) |  |
| Parents’ combined OWOB status |  | 1781 (30.0) |
| Neither OWOB | 1534 (36.9) |  |
| Only father OWOB | 1429 (34.4) |  |
| Only mother OWOB | 423 (10.2) |  |
| Both OWOB | 770 (18.5) |  |
| Parents’ combined Obesity status |  | 1781 (30.0) |
| Neither Obesity | 3484 (83.8) |  |
| Only father Obesity | 313 (7.5) |  |
| Only mother Obesity | 261 (6.3) |  |
| Both Obesity | 98 (2.4) |  |
| Parents’ demographics |  |  |
| Place of residency |  | 0 (0) |
| Södra Lappland (rural) | 619 (10.4) |  |
| Skellefteå (hybrid rural/urban) | 1835 (30.9) |  |
| Umeå (urban) | 3483 (58.7) |  |
| Parents’ socio-economic factors |  |  |
| Parents' country of birth |  | 33 (0.6) |
| Both born in Sweden | 5178 (87.7) |  |
| One born in Sweden | 594 (10.1) |  |
| Both born abroad | 132 (2.2) |  |
| Parents' education |  | 3 (0.01) |
| Both more than a high-school education | 2035 (34.3) |  |
| One more than a high-school education | 1982 (33.4) |  |
| Neither more than a high-school education | 1917 (32.3) |  |
| Family income |  | 1 (0.0) |
| Quintile 1 (Highest) | 1190 (20.0) |  |
| Quintile 2 | 1191 (20.1) |  |
| Quintile 3 | 1187 (20.0) |  |
| Quintile 4 | 1184 (19.9) |  |
| Quintile 1 (Lowest) | 1184 (19,9) |  |
| Parents’ lifestyle factors |  |  |
| Parents’ alcohol habits |  | 18 (0.3) |
| Both non-risky habits | 5279 (89.2) |  |
| One with risk use or addiction | 537 (9.1) |  |
| Both with risk use or addiction | 103 (1.7) |  |
| Parents’ smoking habits |  | 694 (11.7) |
| None smoke | 4715 (89.9) |  |
| One smoke | 497 (9.5) |  |
| Both smoke | 31 (0.6) |  |
| Parents’ snuff habits |  | 552 (9.3) |
| None use snuff | 3767 (70.5) |  |
| One use snuff | 1520 (28.2) |  |
| Both use snuff | 98 (1.7) |  |
| Parents’ drug use |  | 23 (0.4) |
| Both never used drugs | 4946 (83.6) |  |
| One used drugs once or more | 760 (12.9) |  |
| Both used drugs once or more | 208 (3.5) |  |
| Parents’ sleeping habits |  | 137 (2.3) |
| Both 7hrs or more/day | 3978 (68.6) |  |
| One less than 7hrs/day | 1548 (26.7) |  |
| Both less than 7hrs/day | 274 (4.7) |  |
| Parents’ physical activity |  | 69 (1.2) |
| None less than 150 min/week | 2966 (50.5) |  |
| One less than 150 min/week | 2130 (36.3) |  |
| Both less than 150 min/week | 772 (13.2) |  |
