# Supplementary material for: Intergenerational patterns of overweight and obesity among parents and preschool children: a population-based study in Sweden 2010-18
Source: Eur J Public Health. 2026 Nov 8;36(6):ckag201. doi: 10.1093/eurpub/ckag201 (PMC13744698; doi:10.1093/eurpub/ckag201)
Supplement: Supplementary file 1 [file ckag201_supplementary_data.zip › ejph-2026-01-om-0044-File006.docx]

| **Parental BMI category combinations** | Child OWOB | | | | Child Obesity | | | |
| --- | --- | --- | --- | --- | --- | --- | --- | --- |
|  | N=4156 | N=3607 | N=3710 | N=3233 | N=4156 | N=3607 | N=3710 | N=3233 |
|  | Crude OR | Crude OR  Preg^b^ | Adj^a^OR | Adj^a^OR  Preg^b^ | Crude OR | Crude OR  Preg^b^ | Adj^a^OR | Adj^a^OR  Preg^b^ |
| **OWOB within parents** |  |  |  |  |  |  |  |  |
| Neither OWOB | 1 |  | 1 | 1 | 1 |  | 1 |  |
| Only father OWOB | 1.45 (1.16-1.81) | 1.40 (1.10-1.79) | 1.43 (1.13-1.80) | 1.44 (1.11-1.86) | 1.65 (0.95-2.88) | 2.16 (1.10-4.22) | 1.26 (0.70-2.27) | 1.60 (0.78-3.29) |
| Only mother OWOB | 1.91 (1.43-2.57) | 1.88 (1.40-2.54) | 1.98 (1.44-2.71) | 2.13 (1.55-2.93) | 1.92 (0.92-4.02) | 3.09 (1.44-6.63) | 1.63 (0.75-3.54) | 3.22 (1.48-7.02) |
| Both OWOB | 2.24 (1.76-2.84) | 2.20 (1.72-2.83) | 2.44 (1.88-3.16) | 2.49 (1.90-3.27) | 2.52 (1.41-4.51) | 3.39 (1.73-6.65) | 1.97 (1.05-3.69) | 2.92 (1.44-5.94) |
| **Obesity within parents** |  |  |  |  |  |  |  |  |
| Neither Obesity | 1 |  | 1 |  | 1 |  |  |  |
| Only father Obesity | 1.64 (1.23-2.20) | 1.61 (1.17-2.22) | 1.74 (1.27-2.38) | 1.67 (1.18-2.35) | 2.16 (1.16-4.06) | 2.75 (1.45-5.23) | 1.90 (0.94-3.83) | 2.35 (1.15-4.81) |
| Only mother Obesity | 1.70 (1.24-2.33) | 1.62 (1.19-2.22) | 1.75 (1.23-2.49) | 1.64 (1.16-2.31) | 1.72 (0.81-3.62) | 1.68 (0.79-3.58) | 1.45 (0.61-3.47) | 1.46 (0.46-3.50) |
| Both Obesity | 2.88 (1.85-4.47) | 2.72 (1.75-4.22) | 3.01 (1.87-4.84) | 2.88 (1.80-4.60) | 4.18 (1.86-9.37) | 4.23 (1.87-9.56) | 3.51 (1.43-8.65) | 3.21 (1.30-7.97) |

^a^Adjusted for sex of the child, and parental demographic, socio-economic and lifestyle factors

^b^ Mothers BMI from early pregnancy
